# Risk factors for impaired surgical wound healing after spinal tuberculosis surgery: a retrospective comparative trial

**DOI:** 10.3389/fsurg.2026.1708168

**Published:** 2026-03-13

**Authors:** Wang Xiaonan, Hou Daojing, He Ying, Zheng Junru, Wang Xing, Luan Haopeng, Wu Yuanyuan, Li Zhe, Shang Qisong

**Affiliations:** 1 Department of Spine Surgery, Shihezi People's Hospital, Shihezi, Xinjiang, China; 2 Department of Nursing, Shihezi University School of Medicine, Shihezi, Xinjiang, China; 3 Department of Spine Surgery, The Sixth Affiliated Hospital of Xinjiang Medical University, Urumqi, Xinjiang, China

**Keywords:** logistic regression, poor wound healing, predictive model, risk factors, spinal tuberculosis

## Abstract

**Objective:**

To investigate the risk factors associated with poor wound healing following surgery for spinal tuberculosis, and to provide a basis for the clinical prevention and management of postoperative wound complications.

**Methods:**

A retrospective analysis was conducted on the clinical data of 176 patients who underwent surgical treatment for spinal tuberculosis at Shihezi People's Hospital, Xinjiang, between January 2020 and June 2024. Based on wound healing outcomes, patients were divided into a poor healing group (*n* = 49) and a good healing group (*n* = 127). General patient information, laboratory indicators, and surgery-related data were collected. Univariate analysis and multivariate logistic regression analysis were used to identify independent risk factors for poor wound healing, and a predictive nomogram was constructed.

**Results:**

Univariate analysis showed that white blood cell (WBC) count, C-reactive protein (CRP), erythrocyte sedimentation rate (ESR), platelet count, hypoalbuminemia, and anemia were significantly different between the two groups (*P* < 0.05). Multivariate logistic regression analysis indicated that WBC count [OR = 4.057, 95% CI (1.730–18.043)], CRP [OR = 1.039, 95% CI (1.008–1.075)], and ESR [OR = 1.461, 95% CI (1.199–1.941)] were independent risk factors for poor postoperative wound healing (*P* < 0.05). The constructed predictive model demonstrated excellent discriminative ability with an AUC of 0.983, indicating good clinical applicability.

**Conclusion:**

Preoperative elevation of WBC count, CRP, and ESR are independent risk factors for poor wound healing after spinal tuberculosis surgery. Clinical practice should emphasize preoperative anti-tuberculosis therapy, nutritional support, and close monitoring of inflammatory markers to reduce the risk of wound complications.

## 1 Introduction

Spinal tuberculosis, an infectious disease caused by Mycobacterium tuberculosis, often leads to severe structural destruction and neurological compromise (1–3). Surgical intervention is essential for debridement, stabilization, and neural decompression, with outcomes having improved significantly in recent years (4–7).

However, postoperative wound healing remains a major and often underappreciated clinical challenge. Patients with spinal tuberculosis are at heightened risk of poor incision healing due to chronic inflammation, malnutrition, and local infective burden (8). Impaired healing can result in infection persistence, reoperation, prolonged recovery, and increased healthcare costs, representing a significant unmet need in current surgical management. Crucially, there is still a lack of validated, individualized tools to preoperatively stratify this risk, which limits targeted optimization and complicates clinical decision-making.

To address this gap, this study aimed to identify independent risk factors for poor wound healing following spinal tuberculosis surgery and to develop a clinically applicable predictive nomogram. By providing an evidence-based tool for preoperative risk assessment, our findings seek to support earlier identification of high-risk patients and guide personalized preventive strategies, thereby bridging a key deficiency in current management and aiming to improve postoperative outcomes.

## 2 Methods

### 2.1 Study design

Retrospective comparative study.

### 2.2 Time and location

This study was conducted at Shihezi People's Hospital, Xinjiang, China, from January 2020 to June 2024.

### 2.3 Participants

Clinical data were collected from 176 patients diagnosed with spinal tuberculosis who underwent anterior or posterior surgical treatment in the Department of Spinal Surgery at Shihezi People's Hospital between January 2020 and June 2024. Based on wound healing outcomes, the patients were divided into a poor prognosis group (*n* = 49) and a good prognosis group (*n* = 127). The study was approved by the Ethics Committee of Shihezi People's Hospital.

### 2.4 Inclusion criteria

1. Patients with a history of tuberculosis, along with clinical, laboratory, and imaging findings consistent with spinal tuberculosis;
2. Persistent low back pain unresponsive to anti-infective therapy; presence of severe or progressive spinal nerve dysfunction; imaging evidence of significant epidural abscess;
3. Radiographic confirmation of single- or double-segment lesions; severe bone destruction leading to spinal instability;
4. Availability of follow-up data for at least 6 months;
5. Complete medical records, including history, physical examination, laboratory tests [complete blood count, liver and kidney function, tuberculosis antibody, erythrocyte sedimentation rate (ESR), and C-reactive protein (CRP)], and imaging studies (x-ray, CT, MRI).

### 2.5 Exclusion criteria

1. Coexisting active tuberculosis (e.g., pulmonary or intestinal tuberculosis);
2. Concurrent spinal infectious diseases other than spinal tuberculosis, such as spinal disc herniation, tumors, infections, or fractures;
3. Involvement of three or more spinal segments;
4. Inability to tolerate surgery;
5. Severe spinal deformity.

### 2.6 Diagnostic criteria

Spinal Tuberculosis Patients with a history or potential history of tuberculosis infection in other parts of the body. Clinical manifestations may include low-grade fever, night sweats, weight loss, localized spinal pain, and percussion tenderness. Preoperative imaging often reveals intervertebral disc destruction, sequestrum formation, paraspinal or intraspinal abscesses, and spinal cord compression. Laboratory tests may show elevated C-reactive protein (CRP) and erythrocyte sedimentation rate (ESR), as well as a positive tuberculin test, suggesting possible active infection. Postoperative pathological examination confirming typical tuberculous granulomatous structures or caseous necrosis further validates the diagnosis of spinal tuberculosis.

Good Wound Healing will be defined as the absence of signs of infection or dehiscence, with timely suture removal, consistent with routine postoperative healing as described in surgical guidelines (e.g., CDC criteria for no SSI).

Poor Wound Healing will be defined as the presence of one or more of the following: erythema, swelling, purulent discharge, wound dehiscence, delayed healing (>14 days), or sinus formation—criteria commonly used in studies on surgical site complications (9, 10).

### 2.7 Surgical management

#### 2.7.1 Preoperative preparation

A systematic collection of medical history and physical examinations was performed for all enrolled patients. Spinal imaging studies, including x-rays (anteroposterior, lateral, and dynamic views of the thoracic or lumbar spine), CT, and MRI, were completed within 3 days after admission to comprehensively evaluate the affected vertebrae, overall condition, and limb function. For patients with hypertension, blood pressure was controlled below 160/100 mmHg; for those with diabetes, target levels were set at fasting blood glucose <8 mmol/L, 2-hour postprandial blood glucose <10 mmol/L, and urine glucose within the range of+to ++. Medications that could interfere with study results (e.g., anticoagulants) were discontinued. Patients were advised to restrict movement to minimize secondary damage caused by spinal instability, and preoperative nutritional support was emphasized. The antituberculosis chemotherapy regimen was as follows: after a preliminary diagnosis of spinal tuberculosis, oral administration of isoniazid 0.3 g/day, rifampicin 0.45 g/day, ethambutol 0.75 g/day, and pyrazinamide 0.75 g/day was initiated for at least 2–4 weeks. Surgery was considered when patients showed improved appetite, resolution of low-grade fever and night sweats, correction of hypoproteinemia, negative chest x-ray and sputum culture for *Mycobacterium tuberculosis*, and laboratory tests indicating CRP ≤ 20 mm/h, ESR ≤ 50 mm/h, or a significant decrease in both. Before surgery, patients were thoroughly informed about their condition, treatment plan, and associated risks, and written informed consent was obtained.

#### 2.7.2 Surgical techniques

##### 2.7.2.1 Anterior approach group

This procedure is commonly used for debridement of spinal tuberculosis lesions. Via an anterior approach to the affected vertebrae, the lesion is directly exposed, allowing clear visualization and thorough removal of necrotic intervertebral discs, sequestra, abscesses, and caseous tissues. It is suitable for cases with vertebral destruction, abscess formation, severe anterior vertebral collapse, or when posterior approach cannot adequately address the lesion. Additionally, intervertebral bone grafting can be performed to restore spinal alignment and stability. Surgical routes included anterior cervical, transthoracic, and retroperitoneal approaches.

##### 2.7.2.2 Posterior approach group

This technique is indicated for lesions primarily located in the posterior vertebral elements and cases accompanied by severe spinal cord or nerve compression. It involves posterior debridement, bone grafting, and internal fixation. It is suitable for posterior lesion clearance, laminectomy, spinal canal decompression, correction of deformities, and cases with fewer affected segments. Through a posterior approach, compression on the spinal cord and nerves is relieved to prevent further neurological damage. Tuberculous lesions in the posterior vertebral elements are removed, and internal fixation is performed simultaneously to stabilize the spine and correct deformities.

#### 2.7.3 Postoperative management

① Intravenous antibiotics were administered within 24 h postoperatively to prevent infection, and nonsteroidal anti-inflammatory drugs (e.g., celecoxib) were used for pain relief. ② Drainage tubes were removed when the drainage volume was less than 30 mL/24 h. ③ Patients were encouraged to gradually ambulate with lumbar brace protection 1–2 days after surgery, depending on their recovery. ④ Standard quadruple antituberculosis therapy was continued orally: isoniazid 0.3 g/day + rifampicin 0.45 g/day + pyrazinamide 30 mg/(kg·day) + ethambutol 15 mg/(kg·day). The total treatment duration was 9–12 months, with regular monitoring of liver and kidney function and adverse drug reactions. ⑤ Spinal x-ray and CT were reexamined before discharge to assess bone graft fusion and the position of internal fixation devices. ⑥ MRI was performed before discharge to evaluate spinal cord decompression and lesion clearance. ⑦ The lumbar brace was used for 3 months to provide mechanical support and promote spinal stability. ⑧ Regular follow-up visits were required after discharge to monitor liver and kidney function and ensure medication safety.

### 2.8 Postoperative outcome assessment

The primary outcome of this study was the healing status of the surgical incision, categorized as either good or poor healing. To ensure objectivity and minimize assessment bias, wound healing was evaluated independently by two attending surgeons who were not involved in the index surgical procedures. Evaluations were conducted at standardized time points: at discharge, during the first postoperative outpatient visit (approximately 2 weeks), and at subsequent follow-ups as necessary.

Assessment was based solely on objective clinical criteria, including the presence or absence of redness, swelling, purulent discharge, wound dehiscence, hematoma, or sinus formation, as well as the time to complete healing (days). These criteria were aligned with our predefined, referenced definitions (see Section 2.6 Diagnostic Criteria). In cases of disagreement between the two initial assessors, a final consensus was reached through discussion or, if needed, adjudication by a third senior spine surgeon. All assessment data, including photographic documentation when applicable, were recorded prospectively in a standardized case report form. Data collected from electronic medical records for analysis also included patient demographics (age, sex, BMI, history of hypertension, history of diabetes, disease duration, ASA classification), laboratory parameters (hypoalbuminemia, anemia, hemoglobin level, white blood cell count, ESR, CRP, platelet count), and surgical variables (surgical approach, operation time, incision length, intraoperative and total blood loss).

### 2.9 Statistical analysis

Statistical analyses were performed using SPSS (version 26.0, IBM Corp., Armonk, NY, USA). Measurement data are presented as mean ± standard deviation (*x¯* ± s), and group comparisons were conducted using independent sample *t*-tests. Count data are expressed as number and percentage [*n* (%)], and group comparisons were performed using the chi-square test. Univariate analysis was first applied to identify candidate risk factors associated with poor wound healing. Variables with *P* < 0.05 in the univariate analysis were then entered into a multivariate logistic regression model to identify independent risk factors. A nomogram was constructed based on the final logistic regression model using the rms package in R software (version 4.3.1). The predictive performance of the model was evaluated in terms of discrimination [using the area under the receiver operating characteristic (ROC) curve (AUC)] and calibration (using calibration curves with 1,000 bootstrap resamples for internal validation). Decision curve analysis (DCA) was performed to assess the clinical net benefit of the nomogram across a range of threshold probabilities. Sensitivity, specificity, and Youden's index were calculated for key individual predictors. Missing data were handled using Multiple Imputation. Predictive Mean Matching (PMM) was applied for continuous variables, and logistic regression imputation was used for categorical variables. Five imputed datasets were generated and pooled for analysis. It should be noted that the model was developed and validated internally within this single-center cohort using bootstrap resampling. External validation on independent, multi-center datasets is warranted in future studies to confirm its generalizability and clinical applicability. A *P*-value <0.05 was considered statistically significant.

## 3 Results

### 3.1 Analysis of participant enrollment

A total of 176 patients who underwent spinal surgery were included in the study. Based on wound healing outcomes, they were categorized into two groups: a poor healing group (*n* = 49) and a good healing group (*n* = 127). All patients completed the study, and no cases were lost to follow-up.

### 3.2 Group allocation flowchart

**Figure 1:**
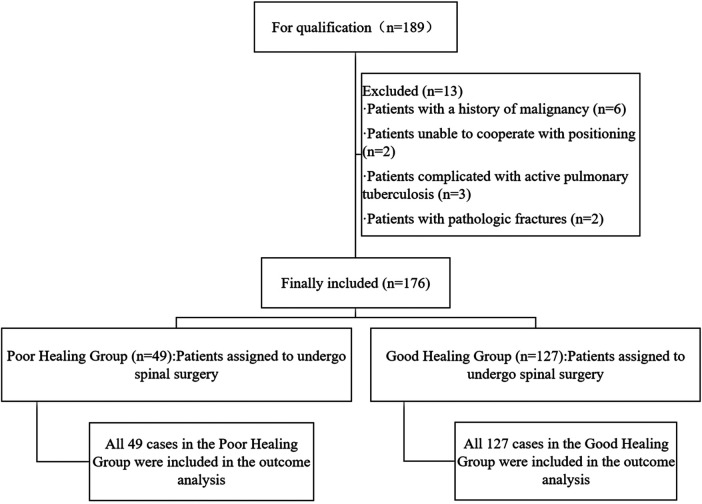
Flow chart of patient assignment.

The flow diagram illustrating patient grouping is shown in Figure 1.

### 3.3 Univariate analysis of postoperative infection following spinal surgery

Univariate analysis revealed that six inflammatory markers—white blood cell (WBC) count, C-reactive protein (CRP), erythrocyte sedimentation rate (ESR), platelet count, hypoalbuminemia, and anemia—were significantly higher in the poor healing group compared to the good healing group (*P* < 0.05). In contrast, other factors such as age, body mass index (BMI), disease duration, operation time, blood loss volume, as well as baseline comorbidities including sex, hypertension, and diabetes, showed no significant differences between the two groups. See Table 1.

**Table 1:** Univariate analysis of factors associated with poor incision healing after spinal tuberculosis surgery.

A. Continuous variable					
Variable	Total	Good healing group (*n* = 127)	Poor healing group (*n* = 49)	*t*	*P*
Age (years)	57.801 ± 9.692	57.718 ± 9.607	58.017 ± 9.907	−0.183	0.855
BMI (kg/m^2^)	22.593 ± 2.330	22.617 ± 2.322	22.532 ± 2.349	0.217	0.828
Course of disease (month)	9.446 ± 3.119	9.354 ± 3.052	9.683 ± 3.275	−0.623	0.534
Hemoglobin (g/L)	132.012 ± 17.723	130.858 ± 18.550	135.001 ± 14.966	−1.390	0.166
White blood cell count (×10^9^/L)	5.903 ± 1.659	5.256 ± 1.232	7.582 ± 1.431	−10.662	<0.001
CRP (mg/L)	43.738 ± 27.256	32.409 ± 18.383	72.869 ± 24.607	−10.337	<0.001
ESR (mm/h)	25.496 ± 11.806	20.136 ± 6.617	39.389 ± 10.942	−11.420	<0.001
Platelet count (×10^9^/L)	290.796 ± 84.649	271.531 ± 68.399	340.727 ± 100.778	−4.388	<0.001
Incision length	11.678 ± 2.244	11.754 ± 2.154	11.480 ± 2.451	0.722	0.471
Intraoperative blood loss	370.073 ± 131.190	360.790 ± 132.512	394.133 ± 124.518	−1.513	0.132
Total blood loss	486.900 ± 153.335	481.653 ± 153.526	500.500 ± 151.998	−0.728	0.468
Operation time	250.618 ± 60.005	252.226 ± 59.631	246.449 ± 60.767	0.570	0.570
Data are presented as mean ± standard deviation. BMI, body mass index; CRP, C-reactive protein; ESR, erythrocyte sedimentation rate.					

B. Dichotomous variable						
Variable	Sorting item	Total	Good healing group (*n* = 127)	Poor healing group (*n* = 49)	*χ* ^2^	*P*
Sex	Female	96 (54.545)	69 (54.331)	27 (55.102)	0.008	0.927
	Male	80 (45.455)	58 (45.669)	22 (44.898)		
Hypertension	No	156 (88.636)	115 (90.551)	41 (83.673)	1.661	0.198
	Yes	20 (11.364)	12 (9.449)	8 (16.327)		
Diabetes	No	163 (92.614)	118 (92.913)	45 (91.837)	0.060	0.807
	Yes	13 (7.386)	9 (7.087)	4 (8.163)		
ASA classify	I	55 (31.250)	37 (29.134)	18 (36.735)	0.951	0.330
	II	121 (68.750)	90 (70.866)	31 (63.265)		
Hypoalbuminemia	No	65 (36.932)	54 (42.520)	11 (22.449)	6.115	0.013
	Yes	111 (63.068)	73 (57.480)	38 (77.551)		
Anemia	No	79 (44.886)	63 (49.606)	16 (32.653)	4.108	0.043
	Yes	97 (55.114)	64 (50.394)	33 (67.347)		
Surgical procedure	Debridement only	116 (65.909)	83 (65.354)	33 (67.347)	0.062	0.803
	Debridement + fixation	60 (34.091)	44 (34.646)	16 (32.653)		
Surgical approach	Anterior	92 (52.273)	71 (55.906)	21 (42.857)	2.413	0.120
	Posterior	84 (47.727)	56 (44.094)	28 (57.143)		

Data are presented as number of patients (*n*) with percentage (%) in parentheses. ASA, American Society of Anesthesiologists.

### 3.4 Multivariate logistic regression analysis of poor wound healing after spinal tuberculosis surgery

**Figure 2:**
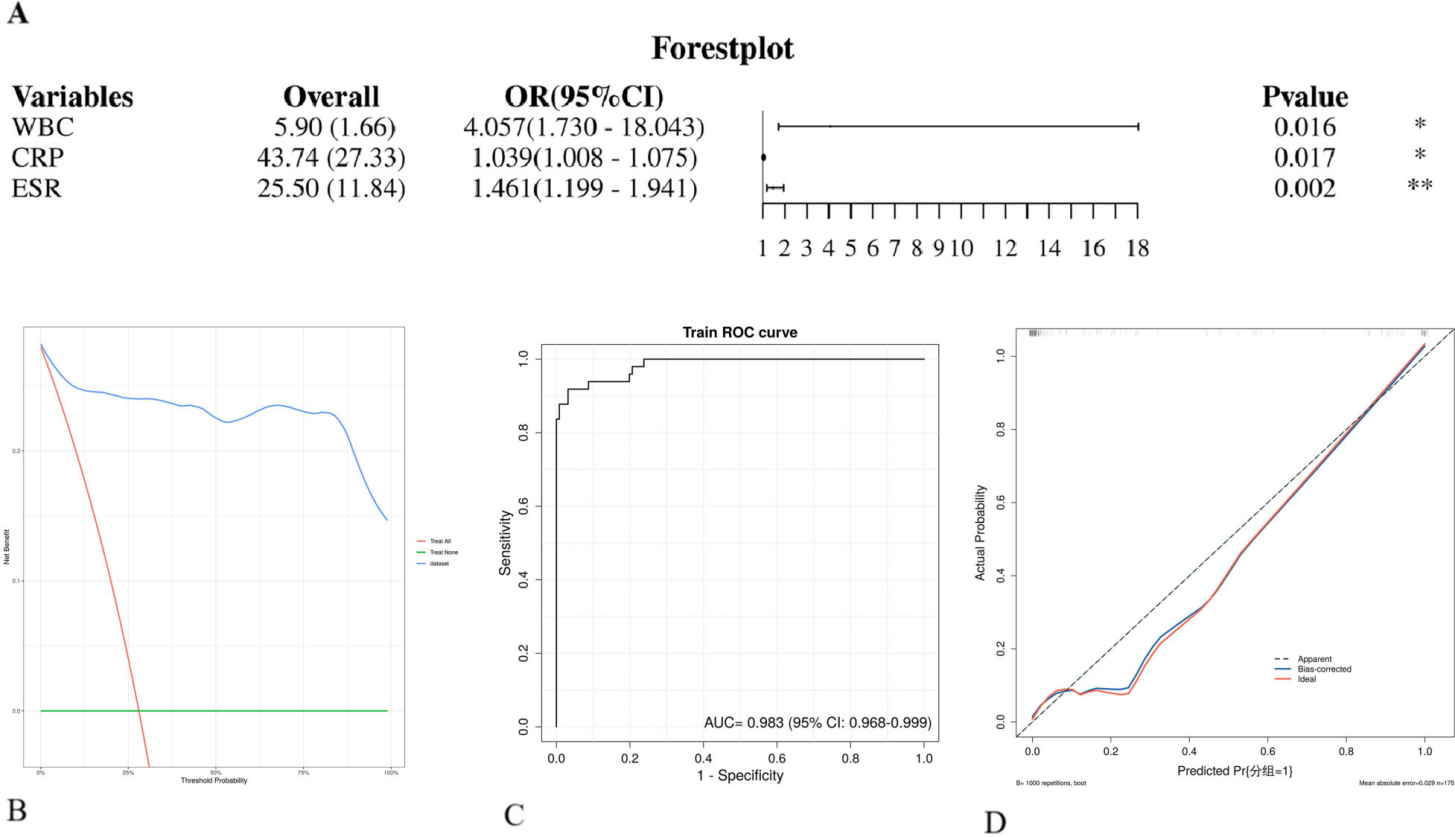
Correlation curves of poor wound healing after spinal tuberculosis surgery based on the nomogram prediction model. **(A)** Forest plot: displays the odds ratios (ORs) and confidence intervals for each variable in the logistic regression model. **(B)** Decision curve analysis (DCA) curve. **(C)** Receiver operating characteristic (ROC) curve of the training set: the area under the curve (AUC) value was 0.983. An AUC closer to 1 indicates better model performance, while an AUC closer to 0 suggests poorer performance. **(D)** Calibration curve: evaluates the calibration of the model by comparing the predicted probabilities with the observed probabilities in the training set.

Using the presence of poor wound healing after spinal tuberculosis surgery as the dependent variable, variables with statistical significance in the univariate analysis (including white blood cell count, CRP, ESR, platelet count, hypoalbuminemia, and anemia) were included as independent variables in the multivariate logistic regression analysis. The results demonstrated that white blood cell (WBC) count [OR = 4.057, 95% CI (1.730–18.043), *P* = 0.016], C-reactive protein (CRP) [OR = 1.039, 95% CI (1.008–1.075), *P* = 0.017], and erythrocyte sedimentation rate (ESR) [OR = 1.461, 95% CI (1.199–1.941), *P* = 0.002] were independent risk factors for poor postoperative wound healing. In contrast, platelet count, hypoalbuminemia, and anemia did not show independent correlations in the multivariate analysis (*P* > 0.05). See Table 2. The predictive model for poor wound healing after spinal tuberculosis surgery, constructed based on the multivariate logistic regression analysis, exhibited excellent predictive performance, with an area under the receiver operating characteristic (ROC) curve (AUC) of 0.983 (95% CI: 0.968–0.999). Decision curve analysis (DCA) further confirmed that the model provided significant clinical net benefit across a wide range of threshold probabilities, demonstrating good clinical applicability for identifying high-risk patients and assisting in treatment decision-making. See Figure 2.

**Table 2:** Multivariate logistic regression analysis of risk factors for poor wound healing after spinal tuberculosis surgery.

Predictor	Estimate	SE	OR (95% CI)	*Z*	*P*
(Intercept)	−22.146	5.069	0.000 (0.000–0.000)	−4.369	0.000
WBC	1.400	0.582	4.057 (1.730–18.043)	2.405	**0** **.** **016**
CRP	0.038	0.016	1.039 (1.008–1.075)	2.377	**0** **.** **017**
ESR	0.379	0.123	1.461 (1.199–1.941)	3.087	**0** **.** **002**
Platelet count	−0.007	0.007	0.993 (0.979–1.005)	−1.052	0.293
Hypoalbuminemia (yes)	0.97	1.094	2.637 (0.324–26.324)	0.886	0.376
Anemia (yes)	−0.931	1.019	0.394 (0.047–2.796)	−0.913	0.361

OR, odds ratio; CI, confidence interval; CRP, C-reactive protein; ESR, erythrocyte sedimentation rate; WBC, white blood cell count.

Bold values represent statistically significant differences (*P* < 0.05).

### 3.5 Development of a nomogram prediction model for poor wound healing after spinal tuberculosis surgery

**Figure 3:**
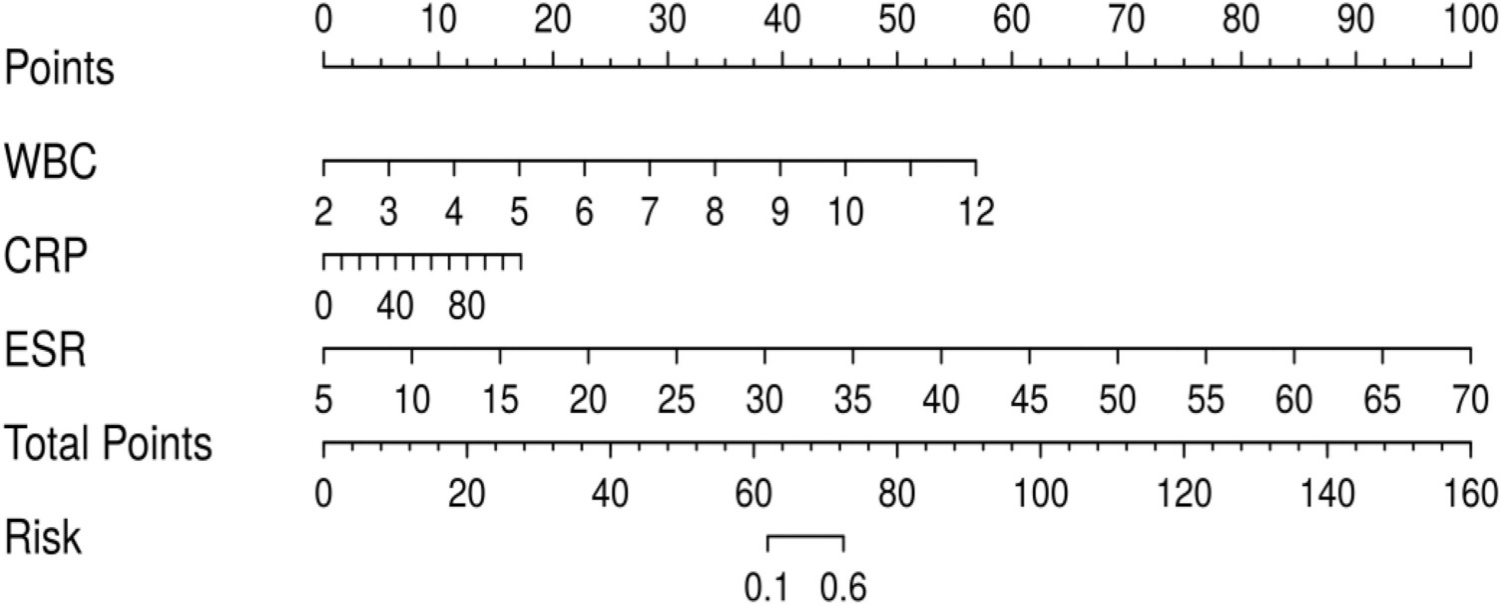
Nomogram predicting the risk of poor wound healing after spinal tuberculosis surgery. CRP, C-reactive protein; ESR, erythrocyte sedimentation rate; WBC, white blood cell count.

A nomogram was developed to predict the risk of poor wound healing following spinal tuberculosis surgery, enabling quantitative and individualized risk assessment. Using a representative patient with a white blood cell (WBC) count of 8.0 × 10⁹/L, C-reactive protein (CRP) level of 50 mg/L, and erythrocyte sedimentation rate (ESR) of 45 mm/h as an example, the corresponding points assigned in the nomogram were approximately 45, 55, and 50, respectively. The total points summed to 150. According to the risk axis of the nomogram, this total corresponded to a probability of approximately 75% for poor postoperative wound healing, indicating that the patient belonged to a high-risk group for postoperative complications. This nomogram serves as a quantitative and individualized tool for clinical risk prediction. See Figure 3.

### 3.6 Sensitivity analysis

**Figure 4:**
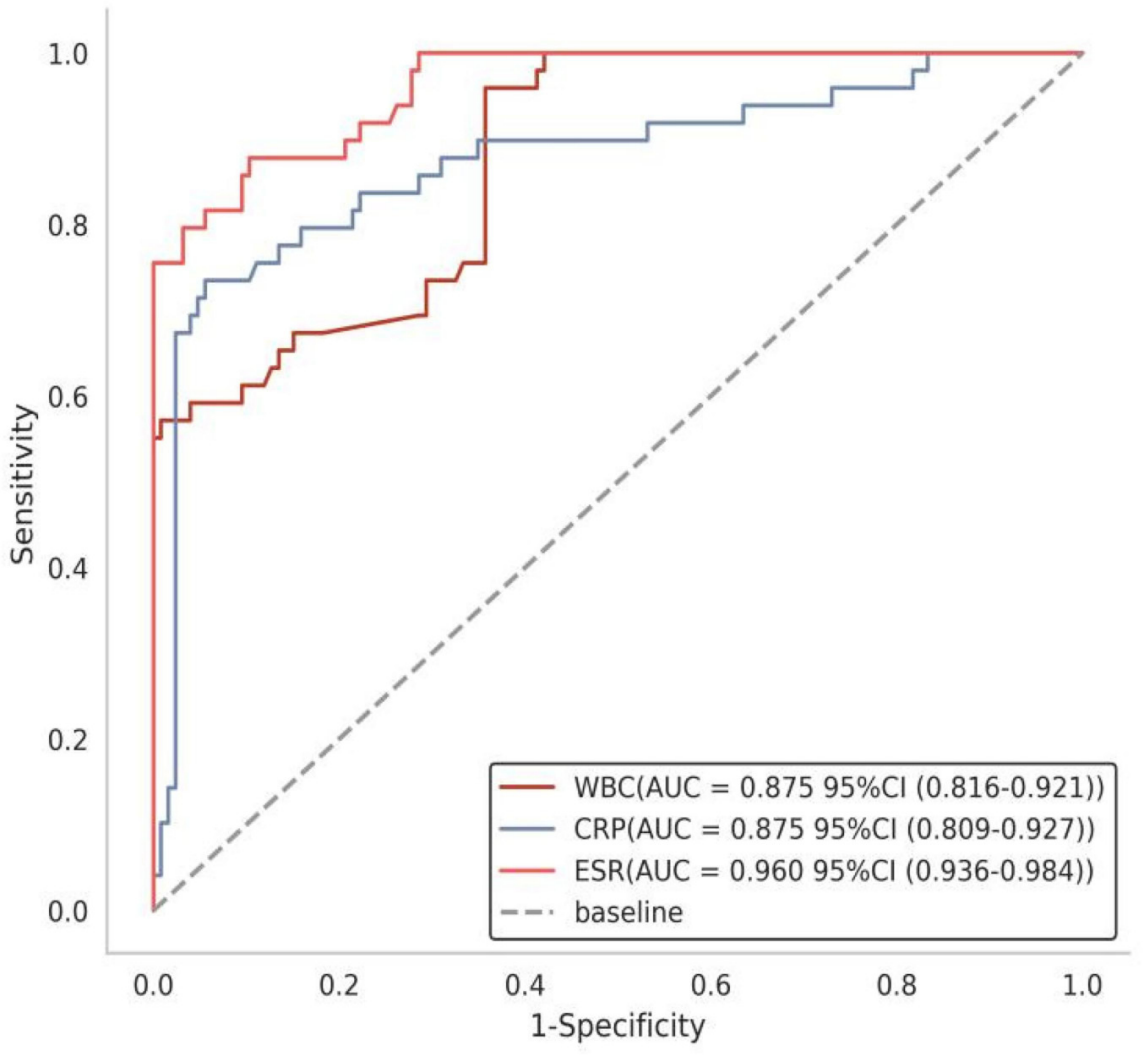
Receiver operating characteristic (ROC) curves of inflammatory indicators for predicting poor wound healing.

The three indicators—erythrocyte sedimentation rate (ESR), C-reactive protein (CRP), and white blood cell (WBC) count—all demonstrated good predictive value for poor wound healing after spinal tuberculosis surgery. Among them, ESR showed the highest predictive performance, with an area under the curve (AUC) of 0.960 (95% CI: 0.930–0.985), a sensitivity of 87.8%, and a specificity of 89.7%. Both CRP and WBC count had an AUC of 0.875; however, CRP exhibited higher specificity (94.4%), while WBC count showed higher sensitivity (95.9%). These results indicate that ESR possesses the best overall discriminative ability, whereas CRP and WBC count excel in specificity and sensitivity, respectively. See Table 3 and Figure 4.

**Table 3:** Sensitivity analysis of indicators for predicting poor wound healing after spinal tuberculosis surgery.

Indicator	AUC (95% CI)	Sensitivity (95% CI)	Specificity (95% CI)	Youden's index (95% CI)	Accuracy (95% CI)
WBC	0.875 (0.820–0.916)	0.959 (0.568–1.000)	0.643 (0.570–1.000)	0.602 (0.553–0.697)	0.778 (0.683–0.912)
CRP	0.875 (0.793–0.930)	0.735 (0.641–0.885)	0.944 (0.813–0.996)	0.679 (0.560–0.804)	0.885 (0.797–0.931)
ESR	0.960 (0.930–0.985)	0.878 (0.727–1.000)	0.897 (0.734–1.000)	0.774 (0.718–0.903)	0.916 (0.814–0.969)

CRP, C-reactive protein; ESR, erythrocyte sedimentation rate; WBC, white blood cell count.

## 4 Discussion

### 4.1 Summary of evidence

Tuberculosis is a major disease that severely impacts global public health, caused by the pathogen *Mycobacterium tuberculosis*. It is one of the oldest diseases in the world and ranks among the top ten causes of death globally, standing as the leading cause of mortality from infectious diseases, far surpassing the alarming death toll of AIDS. Osteoarticular tuberculosis accounts for 1%–3% of all tuberculosis cases, with spinal tuberculosis being the most common form of osteoarticular tuberculosis, representing approximately 50% of cases. Spinal tuberculosis, also known as Pott's disease, was first described by Percivall Pott in 1779 (11). It primarily affects the thoracolumbar segments, often involving the vertebral bodies and intervertebral spaces, while isolated destruction of the posterior elements is rare (3). Moreover, spinal tuberculosis is characterized by an insidious onset, slow progression, and nonspecific clinical symptoms, coupled with a low positive detection rate in *Mycobacterium tuberculosis* culture tests, leading to a high rate of missed or misdiagnosed cases. Additionally, patients with spinal tuberculosis often suffer from malnutrition and compromised immunity, and frequently exhibit poor adherence to anti-tuberculosis drug therapy, all of which contribute to the increased difficulty in treating spinal tuberculosis. Although surgical treatment has significantly improved therapeutic outcomes, complications related to the surgical incision remain a serious concern. The occurrence of incision-related complications greatly diminishes patients' expectations for surgical efficacy and their quality of life (12–14). Therefore, analyzing the factors influencing wound healing and implementing preventive measures are of utmost importance. This study compared relevant hematological indicators between 49 patients with poor wound healing and 127 patients with good wound healing following surgical treatment for spinal tuberculosis. Multivariate logistic regression analysis revealed that elevated white blood cell count, C-reactive protein, and erythrocyte sedimentation rate are independent risk factors affecting wound healing in patients undergoing surgery for spinal tuberculosis.

C-reactive protein (CRP) is a positive acute-phase protein synthesized by the liver and serves as a sensitive inflammatory marker. When the body experiences infection, inflammation, or surgical trauma, changes in CRP occur much earlier than alterations in body temperature or peripheral white blood cell count, making CRP an extremely sensitive indicator of tissue damage and infection (15). A study by Cao Wei et al. demonstrated that dynamic changes in perioperative CRP levels can serve as a monitoring indicator for abdominal incision healing after cesarean section; patients with elevated CRP before and early after surgery had a significantly higher probability of poor wound healing (PWH) compared to those with normal CRP levels. A recent meta-analysis revealed that among tuberculosis patients co-infected with HIV, CRP exhibits high sensitivity and moderate specificity for detecting active pulmonary tuberculosis, indicating its utility in reflecting the risk level of tuberculosis patients and screening for active disease (16, 17). Research by Kang et al. (18). indicated that CRP levels are significantly elevated in patients with active tuberculosis and are closely associated with the severity of the condition. Studies by Kechong et al. (19). showed that elevated CRP levels are correlated with the progression of prostate cancer and may serve as a prognostic predictor for the disease. Pan et al. (20). found that elevated perioperative CRP is an independent risk factor for poor prognosis in epithelial ovarian cancer, particularly in patients with advanced-stage and serous subtypes. The results of this study also suggest that preoperative CRP level can serve as an independent risk factor. We hypothesize that elevated CRP levels indicate a intense systemic inflammatory response, where inflammatory factors and cells significantly interfere with wound healing, and a large number of neutrophils infiltrate the injured site (21). Neutrophil extracellular traps (NETs), which are DNA-protein structures released by neutrophils, act as a defense mechanism to capture and kill pathogens, protecting against microbial invasion in wounds (22). However, NETs have been shown to disrupt the interaction between various cells and factors involved in the wound healing process (23, 24). The DNA and protein components can directly damage peri-wound tissues and disturb the highly coordinated healing process (25, 26).

CRP and ESR are routinely used, sensitive inflammatory markers in clinical practice. Significantly elevated levels directly reflect an active inflammatory response or infection within the patient. For patients with spinal tuberculosis, high levels of CRP and ESR indicate that the inflammatory reaction caused by Mycobacterium tuberculosis is not effectively controlled and the lesions may be in a highly active state (27–29). Performing surgery under such conditions poses an additional challenge, as the body is already in a state of immune stress and hypermetabolism. Surgical trauma can further exacerbate this inflammatory response and disrupt internal homeostasis, thereby severely interfering with key wound healing processes such as fibroblast proliferation and collagen synthesis, ultimately leading to impaired incision healing (30).

An elevated white blood cell count (WBC) typically indicates the presence of infection or a significant inflammatory response. Although tuberculosis is more commonly associated with lymphocyte activity, neutrophils and other immune cells may also be recruited and participate in the immune response within active lesions. A preoperative elevation in WBC suggests that the patient may have concurrent underlying infections (such as bacterial infection) or that the tuberculosis itself has triggered a robust innate immune reaction. This condition can increase susceptibility to postoperative surgical site infection and impair the healing process (31). Our study identified WBC as an independent risk factor, indicating that, in addition to traditional markers such as ESR and CRP, WBC should also be considered a critical component of preoperative assessment (32).

In the univariate analysis, hypoalbuminemia and anemia showed significant differences, yet they did not emerge as independent risk factors in the multivariate analysis. This may be because their effects were masked by more powerful inflammatory markers, or because they exhibited collinearity with the inflammatory state—that is, malnutrition and anemia are themselves consequences of chronic inflammation. However, this does not imply that correcting hypoproteinemia and anemia is unimportant. Adequate nutrition provides the fundamental material basis for tissue repair, and hemoglobin is essential for oxygen delivery; both are necessary conditions for wound healing. They may function as underlying conditions that interact with inflammatory factors to collectively influence the final healing outcome (33).

Based on the three independent risk factors identified above, the constructed nomogram prediction model demonstrated excellent predictive efficacy (AUC = 0.983). The model exhibits strong discrimination and calibration, providing clinicians with an intuitive and quantitative tool for individualized assessment of the risk of poor postoperative wound healing in patients. For example, in patients identified by the model as high-risk, more proactive interventions can be implemented, such as extending the duration of preoperative anti-tuberculosis therapy and nutritional support to ensure inflammatory markers and nutritional status reach a more optimal range for surgery; enhancing intraoperative aseptic techniques and debridement; and closely monitoring the incision postoperatively, prolonging the use of prophylactic antibiotics, while intensifying anti-tuberculosis and nutritional treatments.

Although hypoalbuminemia and anemia showed statistical significance in the univariate analysis, they were not retained as independent risk factors in the multivariate logistic regression model. This may be attributed to several factors. Firstly, inflammatory markers such as WBC, CRP, and ESR, which are closely associated with systemic inflammatory response and infection status, may exert a stronger predictive effect, thereby partially overshadowing the independent contribution of nutritional and anemia indicators in the multivariate model. Secondly, hypoalbuminemia and anemia often coexist with chronic inflammatory states; they may be outcomes rather than fully independent predictors of inflammation, meaning that the inflammatory status could partially mediate the adverse effects of malnutrition on wound healing. Furthermore, when multiple highly correlated variables are included in the model, the statistical independence of certain effects may be attenuated, especially in studies with limited sample sizes.

### 4.2 Limitations of the study

This study has several limitations: (i) It is a single-center retrospective investigation with a limited sample size, which may have introduced selection bias; (ii) Certain potential confounding factors—such as smoking history, specific nutritional interventions, and the quality of postoperative care—were not included in the analysis, possibly affecting the comprehensiveness of the results. Notably, variables such as smoking status, standardized wound care protocols, and patient adherence to anti-tuberculosis therapy were not systematically collected due to the retrospective design. Smoking is known to impair microcirculation and delay wound healing through vasoconstrictive and pro-inflammatory mechanisms. Variations in postoperative wound management—including dressing change frequency, use of antiseptic agents, and compliance with aseptic techniques—may also significantly influence healing outcomes. Furthermore, inconsistent adherence to anti-tuberculosis chemotherapy could lead to suboptimal infection control, thereby elevating the risk of wound-related complications. The absence of these data represents a notable limitation that may affect the interpretation of our findings. (iii) Although the prediction model demonstrated good discriminative ability in our cohort, it was validated only internally. External validation in independent, multi-center populations is necessary to confirm its generalizability and clinical utility.

### 4.3 Conclusion

In summary, this study confirms that elevated preoperative WBC, CRP, and ESR are independent risk factors for poor incision healing following spinal tuberculosis surgery. These findings underscore the critical importance of thorough preoperative preparation, including adequate-course anti-tuberculosis therapy to effectively control active inflammation, and proactive nutritional support to improve the patient's baseline condition. In clinical practice, close monitoring of these markers should be implemented, serving as key references for evaluating surgical timing, predicting operative risks, and formulating individualized treatment strategies. The nomogram model developed in this study facilitates early identification of high-risk patients and provides a valuable tool for guiding precision interventions and improving surgical outcomes.

## Data Availability

The raw data supporting the conclusions of this article will be made available by the authors, without undue reservation.
